# Family history of older adults with Alzheimer's Disease by gender features in a Chinese community setting

**DOI:** 10.1002/alz70860_107164

**Published:** 2025-12-23

**Authors:** Zhaohua Huo, Yip Man Tsang, On Ting Man, Wai Chi Chan, Allen Lee, Linda Lam

**Affiliations:** ^1^ The Chinese University of Hong Kong, New Territories, Hong Kong SAR, China; ^2^ The University of Hong Kong, Hong Kong Island, Hong Kong SAR, China

## Abstract

**Background:**

Understanding the family history of Alzheimer's disease (AD) is helpful in optimal disease prediction and management. This study aims to explore whether gender differences were presented in the family history of older adults with Alzheimer's disease in a Chinese community setting.

**Method:**

Sixty‐nine older adults (age range: 61‐98) diagnosed with mild or major neurocognitive disorder of AD or mixed AD and vascular types were recruited from a community‐ and population‐based prevalence study in Hong Kong. The standardized instrument, Uniform Data Set 3.0, was used to investigate the disease history of cognitive impairment and other neuro‐conditions (e.g., stroke, Parkinson's disease and psychiatric disorders) among participants' parents, siblings and children. All interview questions were self‐reported by participants or their family caregivers. Descriptive analysis was performed after sample weighting, and gender differences were examined by Chi‐square and multi‐variable tests.

**Result:**

As for parents, 26.6% (male vs female: 23.3% vs 30.0%), 7% (6.7% vs 7.7%) and 1.5% (3.4% vs 0.0%) of AD participants reported their parent(s) had conditions of cognitive impairment (CI), stroke and psychiatry disorders, respectively. No significant gender differences in AD participants were shown in parental history (*p* >0.25). As for siblings, 11.1% (male vs female: 10.3% vs 12.5%), 8.8% (3.3% vs 12.8%) and 6.6% (0% vs 12.5%) of participants reported their sibling(s) had CI, stroke and psychiatry disorders. AD females showed higher trends of stroke and psychiatric disorders in their siblings (*p* <0.10). Finally, 7.1% of participants reported their child(ren) had psychiatry disorders, and AD females had a significantly higher report‐rate compared to AD males (12.5% vs 0%) (*p* <0.05).

**Conclusion:**

While nearly 40% of Chinese community‐dwelling older adults with AD indicated a family history of neurocognitive and psychiatry disorders in their close kinships, genders may further explain the genetic and pathological path, especially in other neurologic (e.g., stroke) and psychiatric (e.g., depression, psychosis) conditions.

